# 肺结节的处理策略研究应用新进展

**DOI:** 10.3779/j.issn.1009-3419.2013.05.08

**Published:** 2013-05-20

**Authors:** 虓俊 葛

**Affiliations:** 200040 上海，复旦大学附属华东医院放射科 Department of Radiology, Huadong Hospital Affiliated to Fudan University, Shanghai 200040, China

随着多层螺旋CT的普及应用和肺癌筛查的广泛开展，肺结节的检出率明显提高。对于临床偶然发现的肺结节，多依据其形态学特征、强化方式和强化程度，FDG-PET代谢水平等综合评估其性质。对恶性可能较大的肺结节，多建议经活检或手术获取病理诊断；对性质暂不明确，或良性可能性大，或结节虽有恶性可能但进展缓慢、穿刺活检困难、手术风险大、拒绝创伤性检查者，可通过胸平片或低剂量CT随访，观察病灶动态变化。尽管胸片也可用作肺结节的随访工具，但CT可重复性好、分辨率高、性价比高而更常用。研究提示CT检查中的电离辐射也是潜在的致癌因素之一，而随访可能是漫长、多次的重复性检查，因此确定肺结节随访中合理的CT检查剂量和随访时间间隔十分重要。回顾国内外新近文献，本文对肺结节的随访方案和处理策略进行综述。

## 1 肺结节的分类

肺结节多指胸部平片或CT发现的位于肺部，直径≤30 mm的圆形或类圆形的密度增高影，边界清晰或不清晰^[1]^。依据病灶的数目，可分为单发肺结节即孤立性肺结节（solitary pulmonary nodule, SPN）和多发性肺结节（multiple pulmonary nodule, MPN）。依据结节的密度均匀与否又可分为实质性结节（solid nodule）和磨玻璃结节（ground glass nodule, GGN），后者又包括混合磨玻璃结节（mixed ground-glass nodule, mGGN）及纯磨玻璃结节（pure ground-glass nodule, pGGN）^[2]^。

## 2 肺结节的随访方法

### 2.1 肺结节低剂量CT扫描参数和重建图像算法

定期随访是对因各种原因暂时不能获得定性诊断的肺结节的临时性变通处理，低剂量CT扫描是肺结节随访的理想方法。目前降低CT辐射剂量的方法包括增大螺距、降低管电压、降低管电流、采用迭代重建算法等，但螺距加大、扫描时间缩短时，可能会遗漏表现为磨玻璃密度的小病灶；管电压降低会致X线质量降低，使图像质量降低，因此实际工作中主要通过降低管电流来降低辐射剂量。有研究^[3]^提示30 mAs-50 mAs为最佳的低剂量，若扫描剂量降到20 mAs时，可因胸廓入口骨骼硬化伪影而影响肺尖病灶的检出。另有研究^[4]^认为120 kV、30 mA为肺结节筛查的最佳参数，但对于pGGN和mGGN而言，辐射剂量过低会使图像产生额外的噪声，出现类似磨玻璃密度的病变，而影响原有病灶的显示^[5]^。Funama等^[6]^的研究也提示不合适的低剂量会影响pGGN的检出，如用45 mAs时有25.8%的病灶漏诊，用21 mAs时漏诊率可达39.5%。同时有研究^[7]^指出，在相同的CT剂量下不同的体质量指数所得到的图像质量也不同，对于体质量指数较大的患者应适当增加辐射剂量。Koyama等^[8]^通过对5种不同管电流对肺部GGN进行扫描，并采用标准算法和高分辨率算法进行重建，结果表明重建算法也是影响肺部GGN检出率的一个重要因素，在管电流低于25 mAs扫描采样基础上用标准算法重建时肺结节的显示率明显降低。因此，肺结节随访中应综合考虑患者情况和结节的密度选择合适的低剂量扫描参数，并用标准算法和高分辨率算法进行图像重建。

### 2.2 肺结节随访中生长或变化的评价指标

每次患者复查时都应以首次CT检查的图像为基线进行比较，仔细观察病灶的细微动态变化。有的病灶生长缓慢，尤其是pGGN和部分mGGN，病变的微小变化容易忽略，故需要一种合适的测量方法。通常可借助测量病灶直径和观察密度变化来评估其生长情况，但这种方法不适于直径较小、密度较低、生长较为缓慢的肺结节。研究表明测量病灶的体积优于测量病灶的直径，但此方法多用于实质性肺结节。在部分恶性进展的pGGN和mGGN的随访过程中，可发现病灶的体积不变甚或缩小，而其内的实质成分却增多，表现为病灶的密度增高^[9]^。de Hoop等^[10]^的研究表明，通过测量结节的质量（结节质量=结节体积×结节密度）可以较早发现病灶的生长变化，此方法不仅适用于实质性结节，也适用于pGGN和mGGN，但这种测量方法较为复杂。Zhang等^[11]^对pGGN的研究表明，病灶内的实性成分的含量与其CT密度正相关，因此通过测量CT值可以反映病灶的生长变化，即pGGN的CT值每增加100 HU则肿瘤体积增大10%。

总之，每次患者复查CT时都应详细记录肺结节的数目、位置、大小、密度及形态学等动态信息，并强调尽可能在相同的机型、扫描条件、窗宽、窗位及测量软件下对病灶的生长变化情况进行评估，对于随访中考虑有生长恶变的肺结节，应及时获取病理诊断依据。

## 3 肺结节的随访时间间隔

对于无症状的肺结节患者，选择合理的随访时间间隔，不仅可动态观察病变发展变化，提供处理意见和评估患者预后，还可避免患者接受过多的X线辐射，并节约有限的医疗资源。

Ost等^[12]^建议对于低度恶性的肺结节，应在3、6、12、18、24个月时复查CT；美国呼吸学会^[13]^建议对孤立性肺结节的随访时间为3、6、12及24个月，两者均未对不同大小的肺结节的随访时间间隔做出区别。研究^[14]^表明肺结节的良恶性与其大小有关，大结节的恶性率高于小结节。对于无其它部位原发恶性肿瘤病史的患者， < 5 mm的肺结节恶性率低于1%^[14]^，但8 mm的结节的恶性率可达10%-20%^[15]^。Midthun等^[16]^的研究表明无原发恶性肿瘤病史的患者， < 3 mm结节恶性率约0.2%， < 5 mm肺结节恶性率低于1%，4 mm-7 mm肺结节的恶性率约0.9%，8 mm-20 mm肺结节的恶性率约18%， > 20 mm肺结节的恶性率约50%。另有研究^[14]^结果提示对 < 5 mm的非钙化肺结节，12个月复查与短期复查相比并未延迟患者诊断，对患者预后无明确影响，但5 mm-9 mm的结节6%在4个月-8个月复查时发现病灶有增大。

2005年美国Fleischner学会制定的肺结节的随访指南，就是根据结节的大小结合患者的年龄、肺癌危险因素（如吸烟、被动吸烟、有害气体、电离辐射等）等确定随访时间间隔^[17]^。该指南推荐年龄 > 35岁、无已知肿瘤病史的肺结节患者，经临床风险评估后根据病灶大小决定最初随访时间，再根据病灶的进展情况调整随访时间间隔（表 1，表 2）。随着肺结节研究的深入，尽管指南将病灶大小等因素考虑在内，但主要是针对实质性肺结节。近年呼吸科、胸外科和影像科更关注pGGN和mGGN，Henschke等^[18]^的研究表明，CT筛查中约18%的pGGN为恶性，约63%的mGGN为恶性，mGGN的恶性率甚至高于实质性肺结节，且表现为mGGN，尤其是pGGN，病变多仍处于早期，及时合理的治疗将明显改善患者的预后。2011年肺腺癌国际多学科分类中的原位腺癌（adenocarcinoma *in situ*, AIS）、微浸润腺癌（minimally invasive adenocarcinoma, MIA）CT扫描中多表现为pGGN或mGGN，这两类病灶若行手术切除，患者的无症状5年生存率可达100%或接近100% ^[19]^。

**1:** 2005年Fleischner学会关于 < 8 mm的肺结节的随访方案和处理原则 Recommendations for follow-up and management of nodules smaller than 8 mm detected incidentally at nonscreening CT from the Fleischner scocity in 2005

Nodule size (mm) (Average of length and width)	Low-risk patient (Minimal or absent history of smoking and of other known risk factors)	High-risk patient (History of smoking or of other known risk factors)
≤4	No follow-up needed	Follow-up CT at 12 mo; if unchanged, no further follow-up
> 4-6	Follow-up CT at 12 mo; if unchanged, no further follow-up	Initial follow-up CT at 6-12 mo then at 18-24 mo if no change
> 6-8	Initial follow-up CT at 6-12 mo then at 18-24 mo if no change	Initial follow-up CT at 3-6 mo then at 9-12 and 24 mo if no change
> 8	Follow-up CT at around 3, 9 and 24 mo, dynamic contrast-enhanced CT, PET and/or biopsy	Same as for low-risk patient
Nonsolid (ground-glass) or partly solid nodules may require longer follow-up to exclude indolent adenocarcinoma.		

**2:** 肺癌的风险评估 The relative risk for lung cancer

	Low-risk	Moderate-risk	High-risk
Nodule size	< 8 mm	8 mm-20 mm	> 20 mm
Age (year)	< 45	45-60	> 60
Previous history of cancer	No	Yes	Yes
Smoking status	Never	Smoking now, < 1 bag/day	Smoking now, ≥1 bag/day
Quit smoking	Quit smoking ≥7 years	Quit smoking < 7 years	Never quit smoking
Chronic obstructive pulmonary disease (COPD)	No	Yes	Yes
Asbestos exposure	No	No	Yes
Nodule feature	Smooth	Lobulation	Spiculation

对于pGGN、mGGN的随访时间间隔和随访年限，应区别于实质性肺结节。有研究^[20]^表明pGGN的平均倍增时间为813 d，mGGN的平均倍增时间为457 d，明显长于实质性结节的149 d。另有报道认为尽管肿瘤的平均倍增时间约160 d-180 d，但仍有约22%肿瘤的倍增时间 > 465 d^[21, 22]^，随访超过2年未见生长的肺结节则考虑良性可能大^[12]^，但对于GGN的随访时间应长于2年^[23]^，故对于pGGN和mGGN随访时间间隔及随访年限的确定，都应区别于实质性肺结节。

## 4 肺结节的综合处理策略

按照《I-ELCAP肺癌低剂量CT筛查指南》中关于GGN的随访建议，对于 < 5 mm的mGGN或 < 8 mm的pGGN，应在12个月后进行CT复查；对≥5 mm而≤14 mm的mGGN，应在首次CT检查3个月后复查；对 > 15 mm的GGN可活检也可暂不活检，但应严格定期随访。结节的直径为结节长短径的均值，长径是指在最大层面CT图像上测量的最大径，短径为同一CT图像上测量的与长径垂直的最大值。国际早期肺癌行动计（International Early Lung Cancer Action Program, I-ELCAP）和日本抗肺癌协会（Anti-Lung Cancer Action, ALCA）均建议直径 > 10 mm的GGN需要活检或手术切除，直径 < 8 mm的GGN可12个月左右CT随访一次，发现结节增大时则应选择合理的侵入性检查方法或手术切除^[17]^。以上随访指南中虽有关于GGN的随访建议，但仍较粗略。2013年Fleischner学会发表了针对单发或多发GGN的随访计划（表 3）^[24]^，并强调采用薄层低剂量CT随访^[6]^，要点如下：

**3:** Fleischner学会关于CT检查发现的肺内伴实性结节的随访建议 Recommendations for the management of subsolid pulmonry nodules detected at CT: A statement from the Fleischner scocity

Nodule type	Management recommendations	Additional remarks
Solitary pure GGNs		
≤5 mm	No CT follow-up required	Obtain contiguous 1 mm thick sections to confirm that nodule is truly a pure GGN
> 5 mm	Initial follow-up CT at 3 months to confirm persistence then annual surveillance CT for a minimum of 3 years	FDG PET is of limited value, potentially misleading, and therefore not recommended
Solitary part-solid nodules	Initial follow-up CT at 3 months to confirm persistence. If persistent and solid component < 5 mm，then yearly surveillance CT for a minimum of 3 years. If persistent and solid component ≥5 mm，then biopsy or surgical resection	Consider PET/CT for part-solid nodules > 10 mm
Multiple subsolid nodules		
Pure GGNs ≤5 mm	Obtain follow-up CT at 2 and 4 years	Consider alternate causes for multiple GGNs ≤5 mm
Pure GGNs > 5 mm without a dominant lesion (s)	Initial follow-up CT at 3 months to confirm persistence and then annual surveillance CT for a minimum of 3 years	FDG PET is of limited value, potentially misleading, and therefore not recommended
Dominant nodule (s) with part-solid or solid component	Initial follow-up CT at 3 months to confirm persistence. If persistent biopsy or surgical resection is recommended, especially for lesions with > 5 mm solid component	Consider lung-sparing surgery for patients with dominant leision (s) suspicious for lung cancer
GGN, ground glass nodule; These guidelines assume meticulous evaluation, optimally with contiguous thin sections (1 mm) reconstructed with narrow and/or mediastinal windows to evaluate the solid component and wide and/or lung windows to evaluate the nonsolid component of nodules, if indicated. The use of a consistent low-dose technique is recommended. With serial scans, always compare with the original baseline study to detect subtle indolent growth.		

① 对单发≤5 mm的pGGN，通常不需要CT随访。研究表明pGGN的倍增时间约3年-5年，现有的测量方法对于≤5 mm的病灶欠精确，不同的观察者间也存在测量误差，尽管≤5 mm的pGGN可能为不典型腺瘤样增生（atypical adenomatous hyperplasia, AAH），但其生长缓慢甚至几年无变化^[20]^。故对其随访CT不仅花费大，辐射损害大且随访结果不确切^[25]^，且很少有转移癌表现为pGGN^[26]^，故即使患者有胸腔外的恶性肿瘤，单发≤5 mm的pGGN也不需随访。

② 对单发 > 5 mm的pGGN，需在首次CT检查后3个月复查；若病灶持续存在，则应3年内每年复查一次CT。 > 5 mm的pGGN可能为良性病变（如局灶性纤维化、炎症或出血等），也可能为浸润前病变（如AAH、AIS）或浸润性腺癌（如MIA）等。研究^[2, 27]^表明约20%的 > 5 mm的pGGN可为良性病变，但良恶性GGN有较多重叠难以依据形态学做出准确的诊断^[2]^。现阶段除手术外无其它可靠方法能明确pGGN的性质，但无偱证医学证据表明病灶应常规手术切除^[28]^，故此类病灶应定期随访复查。首次检查后3个月复查如病灶消失，可减少患者不必要的焦虑，但在此期间无需用抗生素治疗^[29]^。因 < 10 mm的pGGN多无代谢，PET不能提供更多的诊断信息^[30]^，故不建议PET检查。虽 > 10 mm或有肺癌病史的患者，随访中结节增大的可能性更大^[31]^，但研究^[32]^表明随访复查过程中发现病灶有生长，再行手术治疗，对患者的预后没有影响。随访中若病灶有生长，穿刺活检也可明确病灶性质，但 < 2 cm的病灶诊断率低于65%， < 1 cm者诊断率仅为35%^[33]^，且pGGN不适合经皮肺穿刺，故穿刺活检仅用于因临床有其它疾病不宜手术的患者。

③ 对孤立的mGGN，尤其是病灶内实性成分 > 5 mm的病灶，应高度警惕为恶性。对8 mm-10 mm的mGGN，应行PET/CT检查得到较为准确的术前诊断和分期信息^[34]^；对需手术的患者可行电视胸腔镜手术（video-assisted thoracoscopic surgery, VATS）下的肺段切除或楔形切除^[35]^。尽管实性成分是浸润性腺癌的一个标志^[18, 26]^，且实性成分越大病灶为恶性可能性越大且预后越差^[36]^，但若为AIS或MIA则建议保守治疗，故对于实性成分≤5 mm的病灶，仅定期复查即可。

④ 对多发的 < 5 mm的GGN，应在首次检查后2年、4年时复查CT。若患者原有肺腺癌切除术史现肺内存在AAH，因仍无研究表明 < 5 mm的多发GGN会进展为浸润性腺癌^[28, 37]^，故首次检查后2年、4年复查CT已足够。

⑤ 对多发pGGN中至少有一个病灶 > 5 mm但无主导病灶者，应3个月后复查CT，此后至少3年内每年复查一次。对于主导病灶的定义，一般认为mGGN的实性成分 > 5 mm，或pGGN > 10 mm但伴有胸膜凹陷征或伴空泡征者，可视为主导病变。若为pGGN或实性成分 < 5 mm的mGGN，随访中病变逐渐增大或密度增高，或实性结节有浸润性腺癌的表现者，也可考虑为主导病灶^[24]^。研究^[28]^表明浸润性腺癌更容易发生于较大的病灶，故对于无主导病灶的多发GGN，无论患者是否有吸烟史都应在3年内每年至少复查一次CT。

⑥ 对有主导病灶的多发GGN，首次检查后3个月复查CT，若肺内病灶仍存在，则应行有创伤性检查获得诊断。虽然目前仍不能鉴别多发GGN是多中心原发癌还是肺癌伴肺转移，但研究表明除粘液性支气管肺泡癌外均应手术切除^[28]^，手术切除病灶中8%-22%为多中心原发肺癌^[38]^。因部分患者会出现新的恶性病灶，故术后3年内每年应至少复查一次胸部CT^[39]^。

## References

[1] Hansell DM, Bankier AA, Macmahon H (2008). Fleischner Society: glossary of terms for thoracic imaging. Radiology.

[2] Kim HY, Shim YM, Lee KS (2007). Persistent pulmonary nodular ground-glass opacity at thin-section CT: histopathologic comparisons. Radiology.

[3] Picozzi G, Paci E, Lopez PA (2005). Screening of lung cancer with low dose spiral CT: results of a three year pilot study and design of the randomised controlled trial "Italung-CT". Radiol Med.

[4] Zhang S, Chen W, Wang W (2010). The best scan parameters for low dose CT scan in early lung cancer screening. Zhongguo Yi Liao Qi Xie Za Zhi.

[5] Zwirewich CV, Mayo JR, Muller NL (1991). Low-dose high-resolution CT of lung parenchyma. Radiology.

[6] Funama Y, Awai K, Liu D (2009). Detection of nodules showing ground-glass opacity in the lungs at low-dose multidetector computed tomography: phantom and clinical study. J Comput Assist Tomogr.

[7] Lee JY, Chung MJ, Yi CA (2008). Ultra-low-dose MDCT of the chest: influence on automated lung nodule detection. Korean J Radiol.

[8] Koyama H, Ohno Y, Kono AA (2010). Effect of reconstruction algorithm on image quality and identification of ground-glass opacities and partly solid nodules on low-dose thin-section CT: experimental study using chest phantom. Eur J Radiol.

[9] Lindell RM, Hartman TE, Swensen SJ (2009). 5-year lung cancer screening experience: growth curves of 18 lung cancers compared to histologic type, CT attenuation, stage, survival, and size. Chest.

[10] de Hoop B, Gietema H, van de Vorst S (2010). Pulmonary ground-glass nodules: increase in mass as an early indicator of growth. Radiology.

[11] Zhang L, Yankelevitz DF, Carter D (2012). Internal growth of nonsolid lung nodules: radiologic-pathologic correlation. Radiology.

[12] Ost D, Fein AM, Feinsilver SH (2003). Clinical practice. The solitary pulmonary nodule. N Engl J Med.

[13] Tan BB, Flaherty KR, Kazerooni EA (2003). The solitary pulmonary nodule. Chest.

[14] Henschke CI, Yankelevitz DF, Naidich DP (2004). CT screening for lung cancer: suspiciousness of nodules according to size on baseline scans. Radiology.

[15] Henschke CI, Naidich DP, Yankelevitz DF (2001). Early lung cancer action project: initial findings on repeat screenings. Cancer.

[16] Midthun DE, Swensen SJ, Jett J (2003). Evaluation of nodules detected by screening for lung cancer with low dose spiral computed tomography. Lung Cancer.

[17] Macmahon H, Austin JH, Gamsu G (2005). Guidelines for management of small pulmonary nodules detected on CT scans: a statement from the Fleischner Society. Radiology.

[18] Henschke CI, Yankelevitz DF, Mirtcheva R (2002). CT screening for lung cancer: frequency and significance of part-solid and nonsolid nodules. AJR Am J Roentgenol.

[19] Travis WD, Brambilla E, Noguchi M (2011). International association for the study of lung cancer/american thoracic society/european respiratory society international multidisciplinary classification of lung adenocarcinoma. J Thorac Oncol.

[20] Hasegawa M, Sone S, Takashima S (2000). Growth rate of small lung cancers detected on mass CT screening. Br J Radiol.

[21] Usuda K, Saito Y, Sagawa M (1994). Tumor doubling time and prognostic assessment of patients with primary lung cancer. Cancer.

[22] Winer-Muram HT, Jennings SG, Tarver RD (2002). Volumetric growth rate of stage Ⅰ lung cancer prior to treatment: serial CT scanning. Radiology.

[23] Aoki T, Nakata H, Watanabe H (2000). Evolution of peripheral lung adenocarcinomas: CT findings correlated with histology and tumor doubling time. AJR Am J Roentgenol.

[24] Naidich DP, Bankier AA, Macmahon H (2013). Recommendations for the management of subsolid pulmonary nodules detected at CT: a statement from the Fleischner Society. Radiology.

[25] Park CM, Goo JM, Lee HJ (2010). Persistent pure ground-glass nodules in the lung: interscan variability of semiautomated volume and attenuation measurements. AJR Am J Roentgenol.

[26] Park CM, Goo JM, Kim TJ (2008). Pulmonary nodular ground-glass opacities in patients with extrapulmonary cancers: what is their clinical significance and how can we determine whether they are malignant or benign lesions?. Chest.

[27] Kodama K, Higashiyama M, Yokouchi H (2002). Natural history of pure ground-glass opacity after long-term follow-up of more than 2 years. Ann Thorac Surg.

[28] Kim HK, Choi YS, Kim J (2010). Management of multiple pure ground-glass opacity lesions in patients with bronchioloalveolar carcinoma. J Thorac Oncol.

[29] Khokhar S, Mironov S, Seshan VE (2010). Antibiotic use in the management of pulmonary nodules. Chest.

[30] Cloran FJ, Banks KP, Song WS (2010). Limitations of dual time point PET in the assessment of lung nodules with low FDG avidity. Lung Cancer.

[31] Hiramatsu M, Inagaki T, Inagaki T (2008). Pulmonary ground-glass opacity (GGO) lesions-large size and a history of lung cancer are risk factors for growth. J Thorac Oncol.

[32] Sawada S, Komori E, Nogami N (2009). Evaluation of lesions corresponding to ground-glass opacities that were resected after computed tomography follow-up examination. Lung Cancer.

[33] Shimizu K, Ikeda N, Tsuboi M (2006). Percutaneous CT-guided fine needle aspiration for lung cancer smaller than 2 cm and revealed by ground-glass opacity at CT. Lung Cancer.

[34] Nair VS, Barnett PG, Ananth L (2010). PET scan ^18^F-fluorodeoxyglucose uptake and prognosis in patients with resected clinical stage IA non-small cell lung cancer. Chest.

[35] Kohno T, Fujimori S, Kishi K (2010). Safe and effective minimally invasive approaches for small ground glass opacity. Ann Thorac Surg.

[36] Aoki T, Tomoda Y, Watanabe H (2001). Peripheral lung adenocarcinoma: correlation of thin-section CT findings with histologic prognostic factors and survival. Radiology.

[37] Maeshima AM, Tochigi N, Yoshida A (2010). Clinicopathologic analysis of multiple (five or more) atypical adenomatous hyperplasias (AAHs) of the lung: evidence for the AAH-adenocarcinoma sequence. J Thorac Oncol.

[38] Nakata M, Sawada S, Yamashita M (2004). Surgical treatments for multiple primary adenocarcinoma of the lung. Ann Thorac Surg.

[39] Mun M, Kohno T (2007). Efficacy of thoracoscopic resection for multifocal bronchioloalveolar carcinoma showing pure ground-glass opacities of 20 mm or less in diameter. J Thorac Cardiovasc Surg.

